# A Comparison of the Methods and Surroundings of English and American Dentists

**Published:** 1891-09

**Authors:** Waldo E. Boardman

**Affiliations:** Boston, Mass.


### A COMPARISON OF THE METHODS AND SURROUNDINGS OF ENGLISH AND AMERICAN DENTISTS.[1]

BY WALDO E. BOARDMAN, D.M.D., BOSTON, MASS.

I WILL give a few facts gleaned from observation and practice during several months in the west of England, in the summer of 1889, while in charge of the large and lucrative practice of an American dentist during his absence in the United States.

We have been informed by practitioners in dentistry in foreign countries that their patients will not submit to an elaborate opera-

---

[1] Read before the Harvard Odontological Society, November 29, 1890.

tion upon the teeth and contiguous parts, nor sit in the chair for a greater period than half an hour, on the average, as do Americans, nor allow that degree of punishment in the extraction of roots or broken-down teeth.

Entering upon the discharge of my duties without previous information regarding an English practice and without introduction placed me in some embarrassment, though my coming had been announced and I was cautioned to "deal as gently with patients as you can, for they dread pain in England more than Satan." I endeavored to follow the golden rule.

This practice was composed of an educated, wealthy, refined people, members of Parliament, the clergy, and many retired army and naval officers, with their families, who reside in the west of England, being the gentry of the counties embraced in a radius of thirty-five or forty miles.

I have been told that an Englishman bathes as often as anybody, that he takes pride in having his tub with him wherever he goes; yet he uses the tooth-brush sparingly, and allows his teeth to decay until ready to be "pulled out," and substituted by what he regards as better ones, inasmuch as "false teeth" do not ache; that they take little care of the organs of mastication and place a low value upon them, and only go to the dentist when a tooth aches. It is puzzling to understand how people, cultured, refined, and wealthy, can tolerate so much uncleanliness in the oral cavity.

Personally, I do not share the same feeling which my English brother has regarding the aversion of the English to an extended operation. My experience taught me quite otherwise. I found the average patient agreeable and sociable, willing to submit to a much longer sitting than I could give if advised to have a tooth removed; they did not require an explanation, but acquiesced.

When they place themselves in the chair they presume the dentist to thoroughly understand his profession and will use his best judgment in the case before him. The average English patient knows less than his American cousin regarding dentistry, nor does he desire any reason why it is thus and so; simply do the work and render the account when completed.

If advised to have a plate, whole or partial, they usually accept the advice as for the best, the expense not entering into the calculation as a rule.

Although in nearly all cases they were favorable to the extraction of worthless teeth and roots, yet I saw many mouths with sets of teeth adapted to fit the palate and cover these decaying roots;

I recall the case of a refined and cultivated lady coming to me to have a superior partial denture removed and refitted. It was fastened to the palate so tightly by clinging to the natural teeth that it required several minutes to remove it, the lady remarking that she had worn it for nearly one and a half years without removal, by advice of the dentist who made it; obviously it was placed in the mouth to remain forever, or till repair either of the mouth or plate was required; but she, unassisted, could not have removed it had she so desired.

I will leave undescribed the condition of her mouth and the state of the plate.

Having cleansed the latter and put it into condition for easy removal, and giving the patient some plain advice regarding cleanliness, etc., she departed, most heartily thanking me for my "great kindness." On another occasion a lady was having a superior full denture fitted over the roots of all the sixteen teeth, though they had all been ground to the gums and their canals cleansed and filled, these are ladies of refinement and wealth.

Very few gum sections are used, plain single teeth being preferable, and in most instances there is no gum about the ten anterior teeth. Gold plates, superior and inferior, are usually held in place by a pair of U-shaped gold springs, their free ends being attached on either side by pivots to upper and lower.

In all cases where extraction of roots or teeth was advised, patients were favorably disposed, gas being used in most instances, rarely extracting without an anæsthetic, never giving ether or chloroform, for the laws preclude all dentists from administering these agents; the family physician must be present.

Operations such as the preparation of cavities for fillings were performed in the usual manner with no more trouble than in my own practice.

Many of the operations required the soft fillings rather than gold, for the reason that caries had so far advanced that the weakness of the tooth admitted of no other material.

The fees in a first-class practice average about the same as with us, except that an English dentist charges for every detail, of whatever name or nature, and in this respect is remunerated more liberally than the American.

Another generation will have passed before the English patient will become educated to the requirements of the American standard of excellence. The English prefer an American dentist to any other, their own countrymen, even, not excepted.

All appointments were made by the servant, who entered the name and address in a book kept in the hall for this purpose. A person wishing an appointment examines the book, and seeing what hours are open on the day desired, selects the time most convenient, and has his name and address written on the line indicated. The operator does not meet the patient till fulfiling the engagement. Another feature of this establishment is a white slate which was every morning placed conspicuously in the operating-room, having written in pencil thereon the hour of appointment and the name of patient. One pleasant feature in an English practice is the custom with patients of all classes, on taking their departure from the operating-room, to invariably shake hands with and thank the operator for the services rendered and the kindness shown, notwithstanding he may have inflicted severe pain in the performance of his duties.

